# The History of Ang. Pect.

**Published:** 1889-02

**Authors:** R. L. Bowcock


### THE HISTORY OF A CASE OF ANGINA PECTORIS TREATED WITH NIRO-GLYCERINE.

By R. L. Bowcock, M. D.

On the night of May 10th, 1888, I was called to see Mrs. J., aged 48. I found her in bed in a sitting position, with both hands clasping her head, suffering excruciating pain and with great difficulty in breathing. I could elicit no information from the patient herself at that time, as she was speechless, but upon questioning her daughter I was told that she had been subject to such attacks for nearly 12 years, though at first they were not so severe and were at intervals of some months apart; but in the last year or so they had become much more frequent and severe, and in the last few months they had been coming on as often as every other day, and on the day I was called to see her she had had two attacks. That night I did but little for her save to give her half grain of morphia with atropine, which had no effect. I returned in the morning and found her out of bed, but suffering with a severe pain in the left temple and eye, which she told me had been constant for a year or more. I examined her throat, as she complained of a cough, and it was very much swollen on the left side externally. I found no lesion either about the throat or the heart, which seemed to be perfectly normal in its action. Judging from the nature of the attacks, being so sudden in their onset and being confined principally to the left side of the body, though the pain extended from the top of the head to the very bottom of the left foot and arm,

I diagnosed angina pectoris and left with the patient an ounce bottle of nitrite of amyl which I happened to have in my satchel, and directed her to inhale five drops of it on a handkerchief whenever she had an attack. This gave some relief, but was not successful. I then prescribed Fowler's solution of arsenic in 5-drop doses three times a day, which was continued throughout the threatment. In a few days I put her upon a 1 per cent solution of nitro-glycerine in doses of one drop, diluted with water, every five hours. This greatly lessened the severity of the attacks almost immediately, and also their frequency. But after using this treatment for a month the attacks became as severe as ever, though not so often—viz: one about every four or five days. I decided to continue the treatment but to increase the dose of nitro-glycerine (sol.), which I directed to be given in 2-drop doses every four or five hours; but after a while this too did not answer. As I saw no bad effect from nitro-glycerine I again decided to increase the dose, which finally reached five drops every four or five hours night and day. At this point the attacks were entirely controlled, and it has now been over two months since she has had any return of the trouble, she has not taken the solution of nitro-glycerine for four or five weeks, and the pain which she has endured for more than a year in the left side of her face and left eye has now disappeared.—*Alabama Med. and Surg. Age.*

